# Severe Splenic Rupture After Minor Blunt Trauma in a Patient With an Epidermoid Splenic Cyst: A Case Report

**DOI:** 10.7759/cureus.114860

**Published:** 2026-08-20

**Authors:** Rodrigo Cisneros-Negrete, Thelma M Alba-Hernández, Ana Álvarez-Vázquez, Alicia M Pimentel-Navarro, Adriana E Ricartti-Villagrán, Quitzia L Torres-Salazar

**Affiliations:** 1 Department of Surgery, Hospital General de Manzanillo, IMSS Bienestar, Manzanillo, MEX; 2 Department of Physiology, Instituto Mexicano del Seguro Social, Durango, MEX; 3 Directorate of Teaching, Quality, and Research, Servicios de Salud del Estado de Durango, Durango, MEX

**Keywords:** blunt abdominal trauma, epidermoid splenic cyst, purulent perisplenic collection, splenectomy, splenic rupture

## Abstract

Splenic rupture following minor blunt abdominal trauma may occasionally be accompanied by unexpected perisplenic findings that complicate diagnosis and management. We report the case of an 18-year-old previously healthy male who presented with progressive abdominal pain 10 days after being struck in the left upper abdomen by a soccer ball during a recreational football match. Initial abdominal ultrasonography demonstrated extensive splenic injury and moderate hemoperitoneum but did not identify a focal infectious collection. Emergency diagnostic laparoscopy revealed an American Association for the Surgery of Trauma grade V splenic injury with active bleeding, dense inflammatory adhesions, and an unexpected encapsulated purulent-appearing perisplenic collection, requiring conversion to open splenectomy. Histopathological examination additionally demonstrated an 11-cm epidermoid splenic cyst with chronic subepithelial inflammation and no evidence of malignancy. The postoperative course was favorable, and the patient was discharged on postoperative day two with subsequent uneventful follow-up. The exact nature of the perisplenic collection and its temporal relationship to the traumatic event could not be definitively established. This case highlights the diagnostic complexity of severe splenic injury associated with an unexpected purulent-appearing perisplenic collection and an underlying epidermoid splenic cyst.

## Introduction

Splenic injury is the most frequently affected solid organ following blunt abdominal trauma, accounting for approximately 32-42% of abdominal solid organ injuries [1]. Advances in imaging and nonoperative management have substantially improved splenic preservation; however, high-grade injuries and hemodynamic instability continue to require surgical intervention. Although delayed complications after splenic trauma have been described, infectious complications remain exceedingly uncommon [2].

Splenic abscess is an uncommon clinical entity, with an estimated incidence ranging from 0.05% to 0.7% in autopsy series and approximately 0.012 hospital admissions per 100,000 population annually [3]. Historically associated with mortality rates approaching 70-100% before the antibiotic era, prompt diagnosis and contemporary management have markedly improved outcomes. Predisposing conditions include hematogenous dissemination, infective endocarditis, immunosuppression, diabetes mellitus, splenic infarction, and, less frequently, splenic trauma [4].

The coexistence of high-grade splenic rupture and a purulent perisplenic collection is rarely reported. Importantly, a true splenic abscess, a perisplenic abscess, and an infected post-traumatic hematoma represent distinct entities and should not be considered interchangeable. Following splenic trauma, purulent-appearing collections may represent secondary infection of a hematoma or devitalized tissue; however, in the absence of microbiological or histopathological confirmation, their infectious nature cannot be established with certainty [5,6]. The present case is noteworthy for the unexpected intraoperative identification of a purulent-appearing perisplenic collection in association with an American Association for the Surgery of Trauma (AAST) grade V splenic rupture after apparently minor blunt trauma.

We report the case of an 18-year-old previously healthy male who presented with an AAST grade V splenic rupture following low-energy blunt abdominal trauma, in whom an unexpected purulent-appearing perisplenic collection was identified during surgical exploration [7]. Histopathological examination additionally revealed an 11-cm epidermoid splenic cyst with chronic subepithelial inflammation. This case highlights the diagnostic complexity of interpreting an unexpected perisplenic collection in the setting of severe splenic injury and underscores the importance of distinguishing confirmed findings from possible post-traumatic or inflammatory processes.

## Case presentation

An 18-year-old previously healthy male with no relevant medical, surgical, or family history presented to the ED with progressive abdominal pain. Ten days before admission, he had sustained a minor blunt abdominal trauma after being struck in the left upper abdomen by a soccer ball during a recreational football match. He remained asymptomatic immediately after the event and continued his usual daily activities without seeking medical attention.

Approximately nine days later, he developed diffuse abdominal pain that began insidiously and progressively worsened over the following 24 hours. He self-medicated with a single dose of naproxen, experiencing only partial temporary relief. As the pain intensified and became predominantly localized to the left upper quadrant, he sought evaluation in the ED. A chronological summary of the patient’s clinical course is provided in Table 1.

**Table 1:** Chronological timeline of the patient’s clinical course. AAST, American Association for the Surgery of Trauma

Time	Clinical event	Diagnostic relevance
Day -10	Minor blunt abdominal trauma	Low-energy mechanism followed by delayed presentation of severe splenic injury
Days -9 to -2	Asymptomatic interval	Prolonged symptom-free period makes the timing and evolution of the splenic injury uncertain
Day 0	Abdominal ultrasonography	Extensive splenic injury and hemoperitoneum identified; no focal infectious collection demonstrated
Day 0	Operative exploration	AAST grade V splenic injury with active bleeding, dense inflammatory changes, and an unexpected purulent-appearing perisplenic collection identified
Day 0	Open splenectomy	Definitive hemorrhage control and management of the severely injured spleen
Postoperative period	Histopathological examination	An 11-cm epidermoid splenic cyst with chronic subepithelial inflammation was confirmed; no malignancy identified
Postoperative day 2	Hospital discharge	Clinically stable, afebrile, tolerating oral intake, with adequate pain control and favorable postoperative evolution
One-month follow-up	Outpatient follow-up	No fever, wound infection, readmission, or other postoperative complications

On admission, the patient was alert and hemodynamically stable. He denied fever, nausea, vomiting, or other constitutional symptoms. Physical examination revealed a soft, nondistended abdomen with preserved bowel sounds and generalized tenderness on deep palpation, more pronounced in the left upper quadrant, without rebound tenderness or other signs of generalized peritonitis.

Initial laboratory studies showed mild leukocytosis with neutrophilia, while hemoglobin and platelet counts remained within normal limits. The coagulation profile, renal function, and serum aminotransferase levels showed no clinically relevant abnormalities. A minimal elevation in total bilirubin was observed. The relevant laboratory findings and their corresponding reference ranges are summarized in Table 2.

**Table 2:** Laboratory findings at hospital admission.

Laboratory parameter	Patient value	Reference range
White blood cell count	14.0 × 10³/µL	4.5-11.0 × 10³/µL
Neutrophils	79.50%	40-65%
Hemoglobin	14.3 g/dL	14-16 g/dL
Platelet count	191 × 10³/µL	150-450 × 10³/µL
Prothrombin time	12.1 seconds	9.7-12.7 seconds
International normalized ratio	1.04	0.80-1.20
Activated partial thromboplastin time	28.5 seconds	22.3-35.9 seconds
Serum creatinine	0.90 mg/dL	0.50-1.40 mg/dL
Total bilirubin	1.12 mg/dL	0-1.0 mg/dL
Aspartate aminotransferase	30 U/L	15-50 U/L
Alanine aminotransferase	21 U/L	5-64 U/L

**Figure 1:**
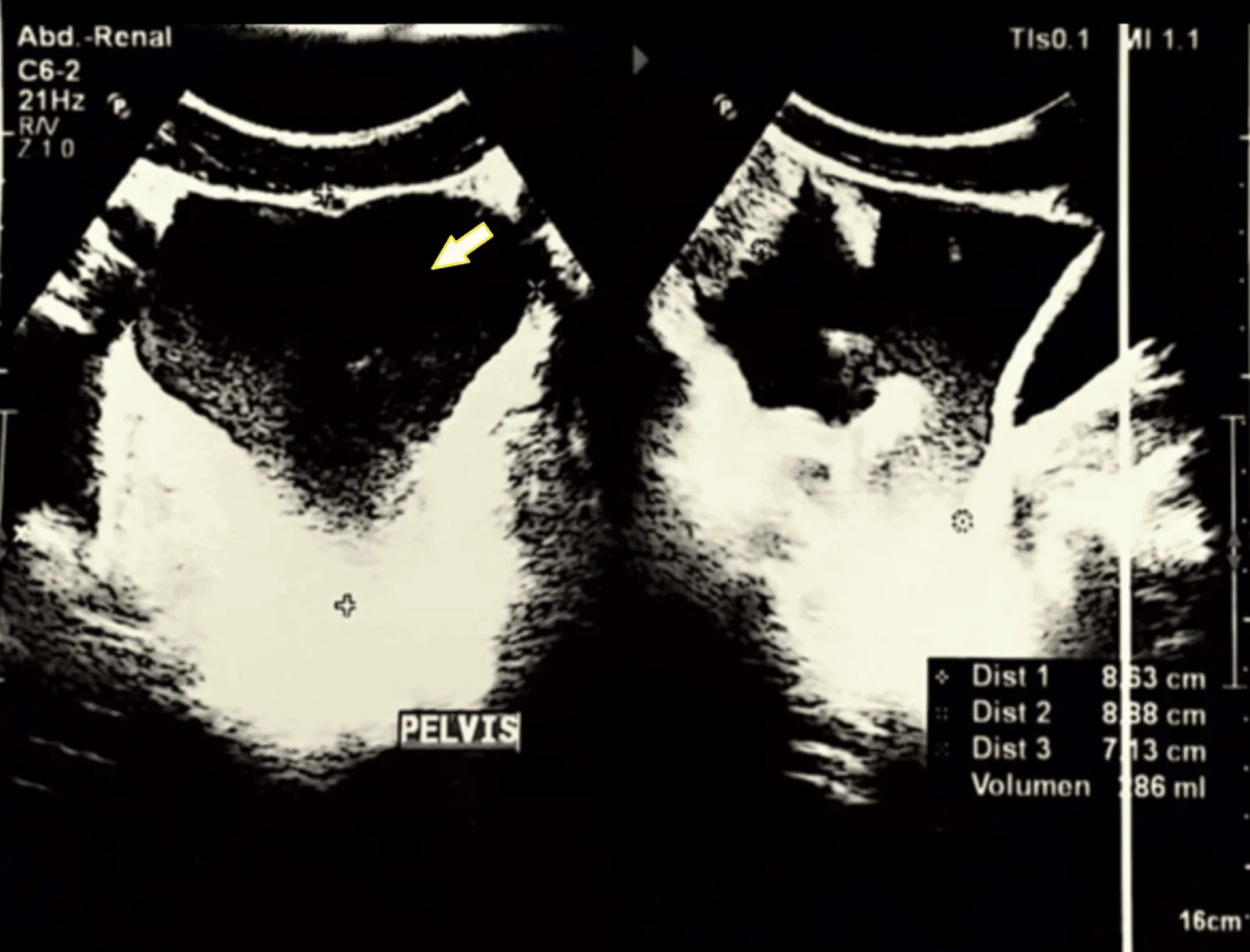
Preoperative pelvic ultrasonography demonstrating free intraperitoneal fluid within the pelvic cavity. The arrow indicates the free fluid collection.

Given the patient’s history of blunt abdominal trauma and progressively worsening abdominal pain, an abdominal ultrasound was performed. The examination demonstrated an extensive splenic laceration, apparently without involvement of the main hilar vessels, together with moderate hemoperitoneum estimated at approximately 500 mL and distributed within the perihepatic, perisplenic, and pelvic recesses. The free intraperitoneal fluid was described as turbid in appearance. No abnormalities were identified in the liver, gallbladder, pancreas, or kidneys. Although the complete ultrasound examination confirmed significant splenic injury, it did not demonstrate a focal infectious collection. Figure 1 illustrates the pelvic component of the free intraperitoneal fluid identified during the ultrasound examination. 

Although the patient remained hemodynamically stable, three serial abdominal examinations demonstrated progressively worsening abdominal pain, increased pain during ambulation, and increasing tenderness on deep palpation. These evolving clinical findings, together with the ultrasonographic evidence of extensive disruption of the splenic architecture and approximately 500 mL of hemoperitoneum, prompted the surgical team to proceed with operative exploration. Splenic angioembolization was not available at the treating institution. Empirical broad-spectrum intravenous antimicrobial therapy with ceftriaxone and metronidazole was initiated because of the turbid appearance of the intraperitoneal fluid and concern for a possible intra-abdominal infectious process. The patient was therefore taken to the operating room for emergency diagnostic laparoscopy with the intention of definitive minimally invasive management if feasible.

**Figure 2:**
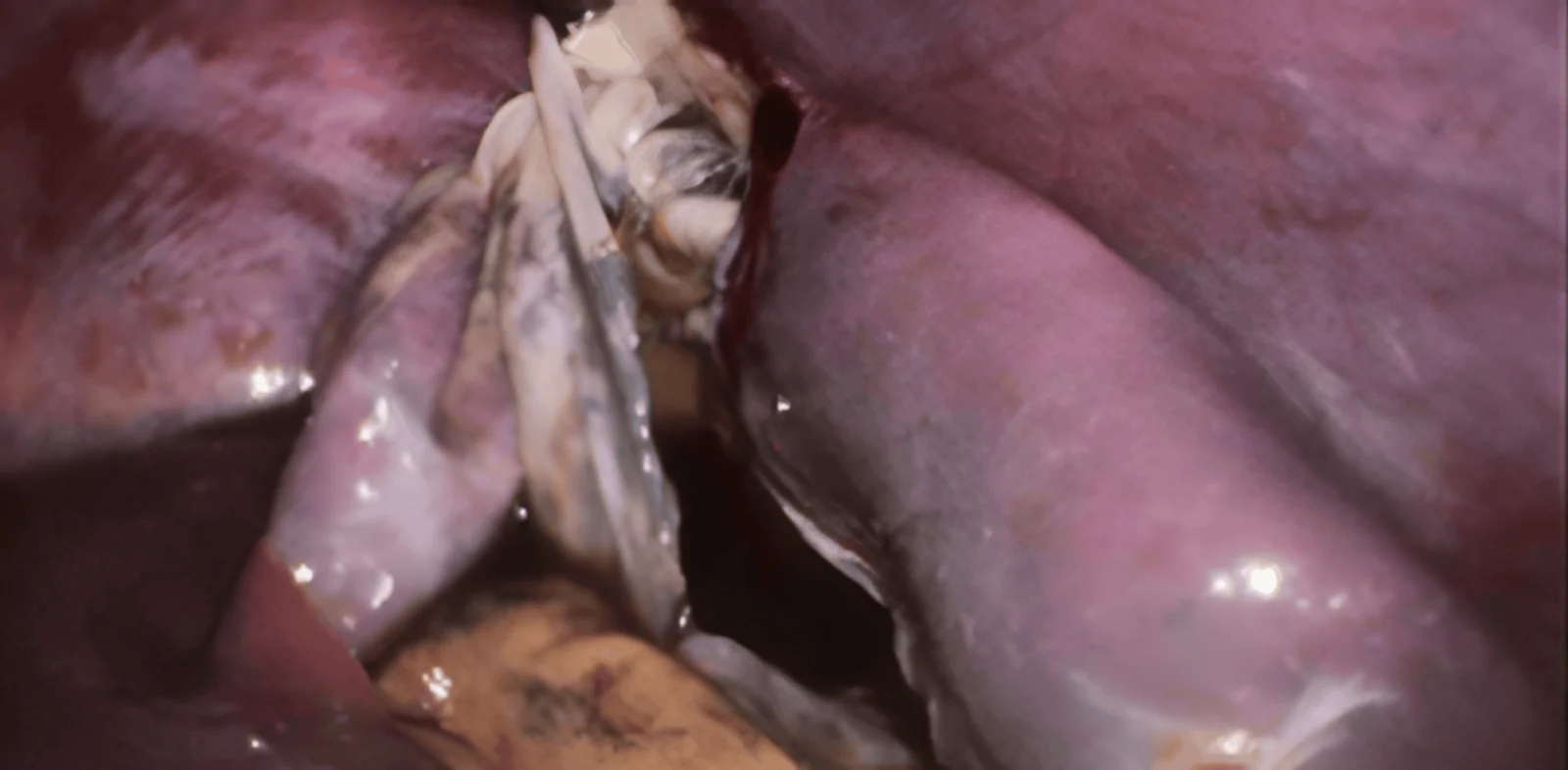
Intraoperative laparoscopic view demonstrating disruption of the splenic parenchyma with capsular discontinuity and exposure of the underlying splenic tissue, consistent with traumatic splenic injury.

Diagnostic laparoscopy revealed approximately 1000 mL of hemoperitoneum composed of fresh blood and organized blood clots, predominantly within the left upper quadrant, together with a marked inflammatory reaction surrounding the spleen (Figure 2). Further exploration demonstrated a shattered spleen with extensive parenchymal disruption, hilar vascular injury, and active bleeding, findings consistent with an AAST grade V injury according to the 2018 Organ Injury Scale [7].

**Figure 3:**
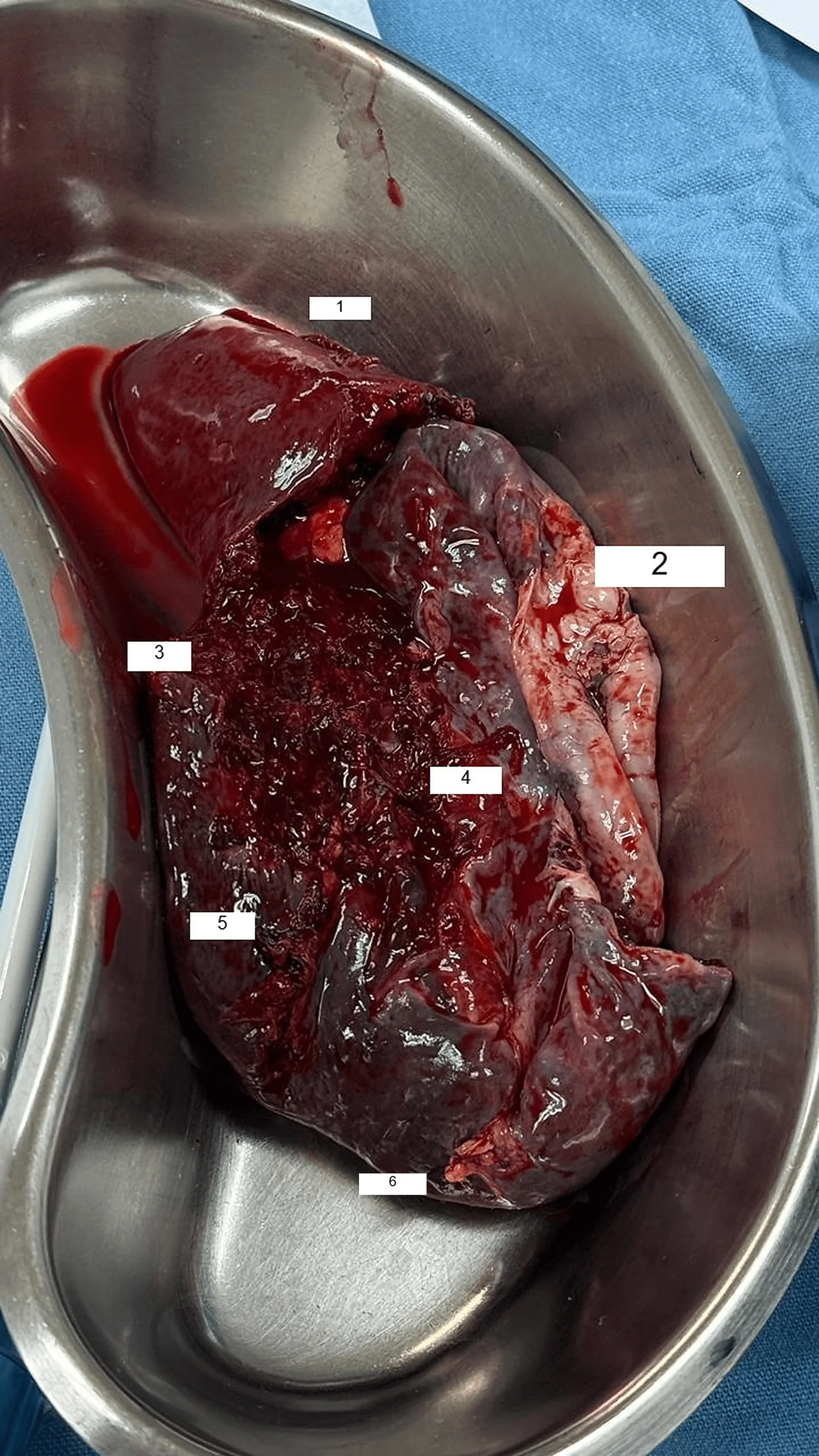
Gross appearance of the splenectomy specimen demonstrating extensive traumatic disruption of the splenic parenchyma. Anatomical landmarks and the main area of rupture are indicated: (1) superior pole, (2) anterior border, (3) posterior border, (4) splenic hilum, (5) rupture zone, and (6) inferior pole.

During careful dissection of the splenic attachments, an unexpected encapsulated perisplenic collection containing approximately 7 mL of thick purulent-appearing material was identified along the superolateral aspect of the spleen, in close contact with the splenic surface and adjacent to the left hemidiaphragm. This finding had not been suspected clinically or detected during the preoperative ultrasonographic evaluation. The combination of extensive splenic destruction, ongoing hemorrhage, dense inflammatory adhesions, and the unexpected perisplenic collection precluded safe laparoscopic management. Conversion to open laparotomy was therefore undertaken to achieve definitive hemorrhage control and facilitate management of the associated perisplenic collection. The gross appearance of the resected spleen is shown in Figure 3.

A total splenectomy was successfully performed, including ligation of the splenic artery and vein as well as the short gastric vessels, followed by copious peritoneal irrigation and placement of a Penrose drain in the splenic bed. Systematic exploration of the remaining abdominal cavity revealed no additional intra-abdominal injuries. A sample of the purulent-appearing material was obtained for microbiological analysis; however, the microbiological results were not available. The splenic specimen was submitted for histopathological examination.

**Figure 4:**
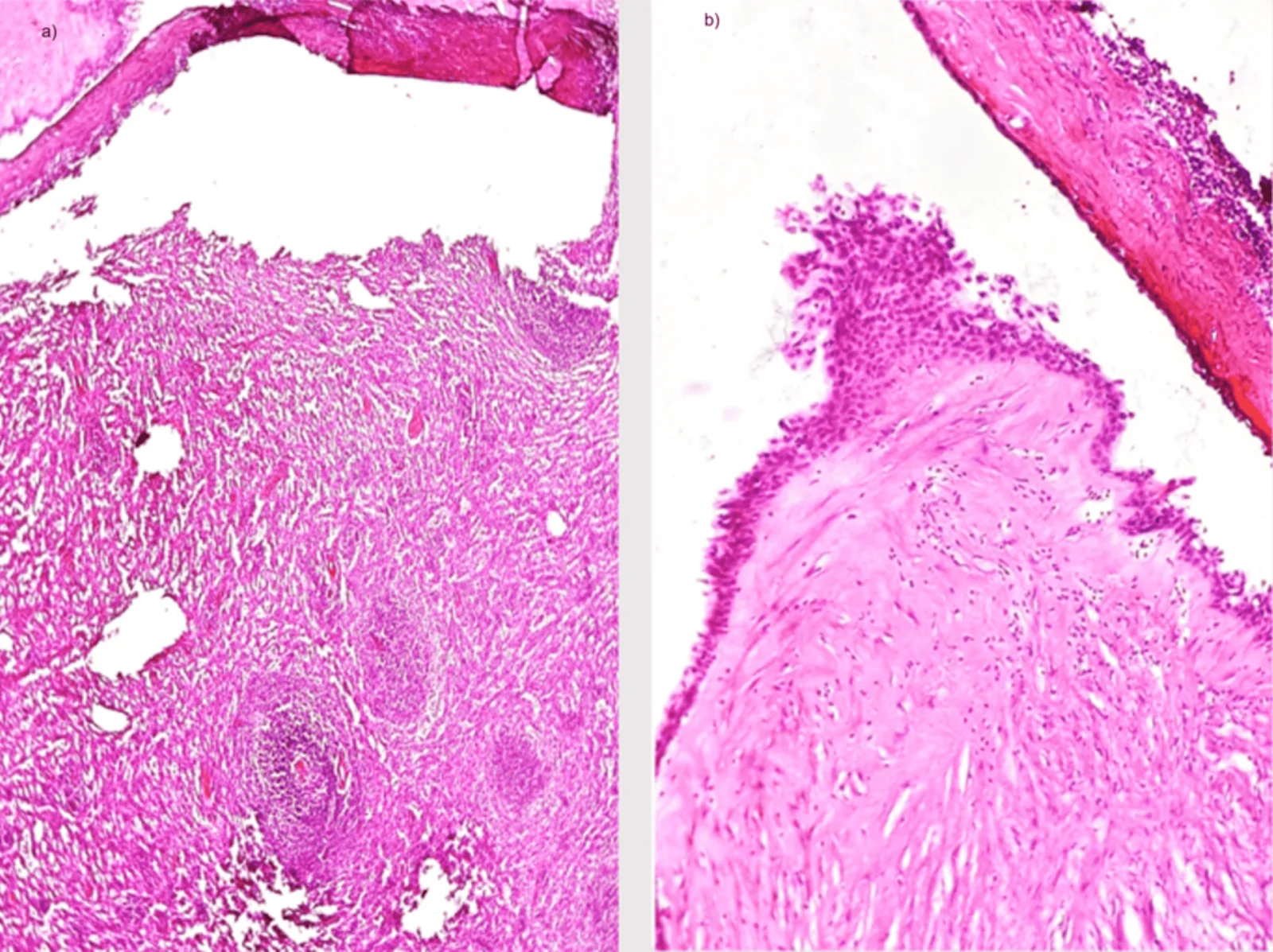
Histopathological findings of the splenectomy specimen (hematoxylin and eosin staining). (A) Cyst wall in continuity with residual splenic parenchyma. (B) Stratified squamous epithelial lining overlying a dense fibrous wall, with chronic subepithelial inflammatory infiltrate and no cytologic atypia. These findings are consistent with an epidermoid splenic cyst.

Histopathological examination of the splenectomy specimen revealed an 11-cm epidermoid (epithelial) splenic cyst. The cyst wall consisted of dense fibrous connective tissue lined by stratified squamous epithelium without cytologic atypia, keratinization, atypical mitotic figures, or cutaneous adnexal structures. A band-like chronic inflammatory infiltrate and focal hematic pigment deposition were identified beneath the epithelial lining. Residual splenic parenchyma was preserved without evidence of infiltrative or neoplastic disease. The final pathological diagnosis was an epidermoid splenic cyst with chronic subepithelial inflammation, negative for malignancy (Figure 4).

The postoperative course was favorable. Broad-spectrum intravenous antimicrobial therapy with ceftriaxone and metronidazole was continued for three days. Estimated intraoperative blood loss was approximately 1200 mL, and the lowest perioperative hemoglobin level was 10.9 g/dL on intraoperative blood gas analysis. The patient remained hemodynamically stable, with a recorded blood pressure of 112/69 mmHg, and did not require vasopressor support. One unit of packed red blood cells was administered postoperatively following assessment by the anesthesia team based on the estimated blood loss and perioperative hemoglobin trend, without transfusion-related complications.

Postoperatively, the patient remained afebrile (36.8°C), with a hemoglobin level of 13.2 g/dL and a white blood cell count of 17.4 × 10³/µL. Oral intake was well tolerated, pain was controlled with analgesia, and active mobilization was maintained. The surgical wound remained well approximated without drainage, exudate, or inflammatory changes. The Penrose drain produced approximately 90 mL of serohematic fluid during the first postoperative 24 hours, subsequently becoming serous with progressively decreasing output; it was then removed.

The patient was discharged home on postoperative day 2 with oral cephalexin 500 mg every eight hours for seven days as empirical continuation therapy in the absence of microbiological susceptibility data. Post-splenectomy immunization against *Streptococcus pneumoniae*, *Neisseria meningitidis*, and *Haemophilus influenzae *type b was scheduled. Outpatient follow-up was performed weekly for one month, with no fever, wound infection, readmission, or other postoperative complications.

## Discussion

Splenic injury is the most common solid organ injury following blunt abdominal trauma, whereas infectious complications involving the spleen remain uncommon [8]. Although trauma has been recognized as a predisposing factor for splenic abscess formation, the coexistence of high-grade splenic rupture and a purulent-appearing perisplenic collection is rarely reported [9]. The present case is particularly noteworthy because an AAST grade V splenic injury was identified after apparently minor blunt trauma and was accompanied by an unexpected purulent-appearing perisplenic collection and a histologically confirmed 11-cm epidermoid splenic cyst. These findings raise several diagnostic considerations, although the nature and temporal origin of the perisplenic collection cannot be established with certainty.

The purulent appearance of the perisplenic collection initially raised concern for an infectious process; however, gross appearance alone cannot establish a diagnosis of abscess. In the absence of available microbiological results, several explanations remain plausible, including secondary infection of a post-traumatic hematoma, an organizing or liquefied hematoma, tissue necrosis, or a sterile inflammatory collection. Post-traumatic splenic abscess has been described after blunt splenic injury, particularly following nonoperative management or splenic artery embolization, where devitalized tissue may become susceptible to secondary bacterial colonization. Tooza and Lee identified only 13 published cases of traumatic splenic abscess, with presentation occurring between 48 hours and four months after injury, illustrating the variable interval during which post-traumatic infectious complications may become clinically apparent [4]. In the present case, the 10-day interval between trauma and surgery is compatible with more than one of these mechanisms and does not permit determination of the temporal sequence.

Histopathological examination provided an additional finding that modifies the interpretation of the case. The splenectomy specimen contained an 11-cm epidermoid cyst lined by stratified squamous epithelium, with a fibrous wall, chronic subepithelial inflammation, and focal hematic pigment deposition, without evidence of malignancy. Epidermoid splenic cysts are uncommon true cysts that may remain clinically silent until incidentally detected or complicated. Ibikunle et al. noted that epithelial splenic cysts may reach considerable size while remaining asymptomatic or producing nonspecific symptoms and emphasized the role of histopathological examination in definitive classification [10]. More specifically, Goai et al. reported spontaneous rupture of a 9-cm epidermoid splenic cyst in a previously healthy young adult, resulting in a subcapsular hematoma and hemoperitoneum and ultimately requiring splenectomy [11]. Histopathological examination in that case confirmed a benign epidermoid cyst with possible partial rupture. In this patient, the cyst therefore provides objective evidence of preexisting splenic pathology; however, neither its causal relationship with the traumatic rupture nor an infectious origin of the perisplenic collection can be established from the available findings.

The clinical and imaging findings further illustrate the diagnostic complexity of this case. The patient remained afebrile and had no overt systemic manifestations of infection, differing from the classic presentation of splenic abscess described by Tooza and Lee and Singh et al., in which fever, leukocytosis, and localized abdominal pain predominate [4,6]. Ultrasonography identified extensive splenic injury and hemoperitoneum but did not identify the perisplenic collection or accurately characterize the hilar vascular injury subsequently demonstrated at surgery. In hemodynamically stable adults with suspected splenic trauma, contrast-enhanced CT provides superior characterization of parenchymal injury, vascular involvement, active bleeding, and associated collections and is central to contemporary decision-making regarding nonoperative management and angioembolization. Podda et al., in the 2022 World Society of Emergency Surgery consensus document, support nonoperative management in hemodynamically stable patients when appropriate monitoring and rescue resources are available and recognize a role for splenic artery embolization in selected patients, particularly those with high-grade injuries or vascular findings [12]. In the present case, operative exploration was undertaken after three serial abdominal examinations demonstrated progressive pain and increasing tenderness in conjunction with extensive splenic disruption and hemoperitoneum on ultrasonography. Splenic angioembolization was not available at the treating institution.

These strategies differ from the management of an isolated splenic abscess, for which antibiotics combined with image-guided percutaneous drainage may preserve splenic function in appropriately selected patients, as demonstrated by Fadul et al. [13]. In the present case, however, operative exploration demonstrated extensive parenchymal disruption, hilar vascular injury, active hemorrhage, and dense inflammatory changes. These intraoperative findings, together with the purulent-appearing perisplenic collection, precluded safe spleen-preserving laparoscopic management, and conversion to open splenectomy provided definitive hemorrhage control and management of the severely injured spleen.

Previous reports illustrate the heterogeneity of splenic infectious and inflammatory complications. Zarei et al. observed that CT commonly established the diagnosis of ruptured splenic abscess in stable patients, whereas operative exploration served both diagnostic and therapeutic purposes in urgent or complex presentations [5]. Komeno et al. described extensive adhesions, hemorrhage, and necrosis in chronic splenic inflammatory disease, demonstrating that marked local inflammatory changes may occur in indolent splenic pathology [14]. These observations provide useful context for the operative findings in our patient but cannot establish the nature or chronology of the perisplenic collection.

The principal limitations of this report are the absence of available microbiological culture results and preoperative contrast-enhanced CT. Consequently, the purulent-appearing perisplenic collection cannot be definitively classified as an abscess, infected hematoma, liquefied hematoma, or sterile inflammatory collection, and its temporal relationship to the traumatic event remains uncertain. Histopathological examination confirmed an epidermoid splenic cyst with chronic subepithelial inflammation but did not establish an infectious etiology. Nevertheless, the operative findings, histopathological documentation, and clinical follow-up provide a detailed characterization of this unusual presentation.

## Conclusions

This case illustrates the coexistence of severe splenic rupture following minor blunt abdominal trauma and an unexpected purulent-appearing perisplenic collection of uncertain nature. Histopathological examination additionally revealed an 11-cm epidermoid splenic cyst with chronic subepithelial inflammation. Although this finding confirmed preexisting splenic pathology, neither the nature of the perisplenic collection nor its temporal relationship with the traumatic event could be definitively established.
